# The Potential of Gut Commensals in Reinforcing Intestinal Barrier Function and Alleviating Inflammation

**DOI:** 10.3390/nu10080988

**Published:** 2018-07-29

**Authors:** Kaisa Hiippala, Hanne Jouhten, Aki Ronkainen, Anna Hartikainen, Veera Kainulainen, Jonna Jalanka, Reetta Satokari

**Affiliations:** 1 Immunobiology Research Program, Faculty of Medicine, University of Helsinki, 00290 Helsinki, Finland; kaisa.hiippala@helsinki.fi (K.H.); hanne.jouhten@helsinki.fi (H.J.); aki.ronkainen@helsinki.fi (A.R.); anna.hartikainen@helsinki.fi (A.H.); jonna.jalanka@helsinki.fi (J.J.); 2 Pharmacology, Faculty of Medicine, University of Helsinki, 00290 Helsinki, Finland; veera.kainulainen@helsinki.fi

**Keywords:** commensal bacteria, intestinal health, next generation probiotics, dysbiosis, intestinal permeability, butyrate producing bacteria, anti-inflammatory

## Abstract

The intestinal microbiota, composed of pro- and anti-inflammatory microbes, has an essential role in maintaining gut homeostasis and functionality. An overly hygienic lifestyle, consumption of processed and fiber-poor foods, or antibiotics are major factors modulating the microbiota and possibly leading to longstanding dysbiosis. Dysbiotic microbiota is characterized to have altered composition, reduced diversity and stability, as well as increased levels of lipopolysaccharide-containing, proinflammatory bacteria. Specific commensal species as novel probiotics, so-called next-generation probiotics, could restore the intestinal health by means of attenuating inflammation and strengthening the epithelial barrier. In this review we summarize the latest findings considering the beneficial effects of the promising commensals across all major intestinal phyla. These include the already well-known bifidobacteria, which use extracellular structures or secreted substances to promote intestinal health. *Faecalibacterium prausnitzii*, *Roseburia intestinalis*, and *Eubacterium hallii* metabolize dietary fibers as major short-chain fatty acid producers providing energy sources for enterocytes and achieving anti-inflammatory effects in the gut. *Akkermansia muciniphila* exerts beneficial action in metabolic diseases and fortifies the barrier function. The health-promoting effects of *Bacteroides* species are relatively recently discovered with the findings of excreted immunomodulatory molecules. These promising, unconventional probiotics could be a part of biotherapeutic strategies in the future.

## 1. Introduction

The human gastrointestinal microbiota is a complex ecosystem consisting mainly of bacteria, but also viruses and eukaryotic organisms. It has been estimated that we harbor 10^13^ bacterial cells, which equals the number of eukaryotic cells in the human body [1]. In total, over 3000 bacterial species have been found in human feces to date [2], although only 1000 of these have been characterized by culturing, while the remaining have only been detected by sequencing [3]. Most individuals have around 200 different species in their digestive tract with highly variable abundance and a combination that makes the microbiota make-up very personal [4]. From this bacterial population about 30% are shared between subjects, creating the so-called common core microbiota [4,5].

The intestinal microbiota affects the human health in many ways and is considered a major contributing factor in a number of diseases. Microbiota plays a role in the maintenance of the intestinal barrier, which is essential for homeostasis and functionality of the gut. Impaired barrier function is associated with both intestinal and systemic diseases. In a balanced situation regarding the pro- and anti-inflammatory properties of the microbiota, it stimulates and challenges the host and its immune system at an appropriate level to keep the defense mechanisms alerted, while, at the same time, providing regulatory signals to induce tolerance towards commensal microbiota.

The digestive tract has two distinct microbial ecosystems, the luminal and the mucosal microbiota [6]. In the luminal compartment, over 90% of bacteria belong to Firmicutes and Bacteroidetes, and the minor phyla include Actinobacteria, Proteobacteria, and Verrucomicrobia. In the mucosal layer, the number of bacteria, as well as the diversity, is considerably lower, and the composition is clearly different as Firmicutes are generally higher in abundance compared to the Bacteroidetes in both humans [7] and mice [8].

Microbial composition is affected by a number of endogenous and exogenous factors, such as hosts’ physiology and immunity, diet, and medication. In contemporary societies, an overly hygienic lifestyle with few exposures to environmental microbes, consumption of processed and fiber-poor foods and frequent use antibiotics are considered as major modulators of human microbiota [9]. Strong perturbations like these may lead to a state of dysbiosis, which has been associated with a number of human diseases [10]. The causal links between microbiota dysbiosis and disease states are currently under intense investigation, as well as the mechanisms by which the microbiota could contribute to the pathological processes [10].

Microbiota dysbiosis is generally characterized by altered composition, as well as reduced diversity and stability [11]. This has also led to observations where the relative proportion of facultative anaerobic bacteria has been increased and the proportion of strictly anaerobic bacteria with protective functions, such as the production of short-chain fatty acids (SCFA) has been reduced [12]. Overall, a dysbiotic microbiota has not only lost the capacity to support intestinal homeostasis, but it may even exacerbate intestinal inflammation [11]. For example, an increase in the gamma-Proteobacteria levels with highly proinflammatory cell wall structures, i.e., lipopolysaccharide (LPS), will induce inflammation and the following oxidative stress, in turn, will exacerbate dysbiosis by promoting facultative anaerobic bacteria [13]. Furthermore, microbiota dysbiosis can impair the epithelial barrier leading to so-called leaky gut allowing the intestinal content to be in contact with the host periphery potentially inducing inflammatory responses, which is often observed in several human diseases [12]. While anti-inflammatory drugs provide different alternatives to dampen inflammation effectively, it would also be essential to treat the microbial dysbiosis and strengthen the epithelial integrity in order to restore intestinal homeostasis in the long-term. The growing knowledge of the importance of the human microbiota suggests that commensal species can be isolated and used for therapeutic purposes as so-called next-generation probiotics, alongside lactobacilli and bifidobacteria with a long history of use as probiotics. Next-generation probiotics are potential novel health-promoting commensal strains, which have not yet been used for nutritional and therapeutic purposes and could possibly be exploited in dietary and pharmaceutical applications. Emerging evidence shows that a large number of commensal species across all major intestinal phyla have the ability to strengthen the epithelial barrier and alleviate inflammation, as will be discussed in the following sections.

In this review we summarize the beneficial effects of well-known, promising commensal bacteria on the intestinal epithelial barrier and discuss their possible use as unconventional, so-called next-generation probiotics of the future. The importance of butyrate-producing bacteria to the intestinal health is well documented, but at the molecular level the mechanisms, e.g., effector molecules secreted by commensal bacteria, are still being investigated. Hence, this review summarizes the potential mechanisms known so far behind the health-promoting action of the commensals in the gut.

## 2. Intestinal Barrier Function

**Figure 1:**
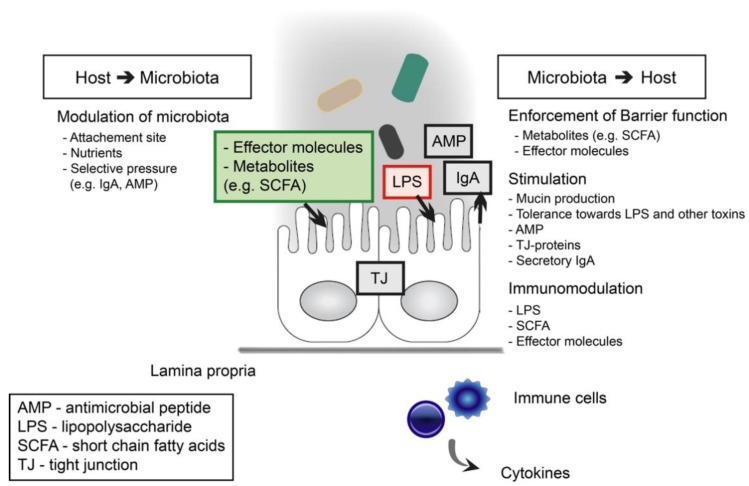
Host-microbiota interactions affecting the epithelial barrier function.

The intestinal epithelium acts as a barrier between the host and the luminal confinement of commensal bacteria, enabling the symbiotic relationship between the two (Figure 1). In addition to the physical barrier of epithelial cells and mucus layer, other host defense mechanisms include the secretion of antimicrobial peptides (AMPs) and secretory immunoglobulin A (sIgA), as well as the presence of immune cells like phagocytes and macrophages [14]. The most prominent antimicrobial peptides include defensins, cathelicidins, and C-type lectins, which are expressed as a result of pattern recognition receptor (PRR) activation in the intestinal epithelial cells [15]. In order to sustain intestinal integrity, the epithelial cells need to be renewed constantly by continuous differentiation from stem cells to goblet cells, Paneth cells, enteroendocrine cells, or enterocytes [16]. Enterocytes form the physical barrier by linking together with different cell junctions, including desmosomes, adherens junctions, and tight junctions. In turn, goblet cells secrete mucin, which forms a thick, protective mucus layer on the epithelial mucosa. Paneth cells are specialized producers of antimicrobial molecules mostly located in the small intestine. Endocrine cells secrete serotonin and various neuropeptides, which act as signals in the crosstalk between the enteroendocrine cells and the immune system. Gut-associated lymphoid tissue (GALT) is located under the epithelial layer, where submucosal dendritic cells (DCs) sample antigens from the lumen or luminal antigens are presented to DCs by microfold cells (M-cells) leading to the activation of IgA secretion [17]. Recently it was discovered that IgA, besides its defensive role, also generates host–microbe symbiosis by enabling mucosal colonization of bacteria [18]. *Bacteroides fragilis* modulates its surface structures to bind IgA, which is needed for the colonization of the mucosal niche in the gut.

Tight junctions, such as claudins and occludin, are intercellular junctions connecting the epithelial cells tightly together and controlling the paracellular permeability [19]. Hence, the tight junction proteins also moderate the transepithelial transport. The intestinal barrier enhancement, for example, by probiotic bacteria, is associated with changes in the tight junction protein expression and distribution. Intestinal inflammation, commensal microbes, and dietary components are among the main factors affecting epithelial permeability [20]. Food factors, including amino acids like glutamine and tryptophan, as well as polyphenols, enhance the barrier integrity. Glutamine, a well-studied food component, is a primary energy source for enterocytes, which regulates tight junction expression and localization in Caco-2 intestinal cells [21]. On the other hand, there are also detrimental food factors, such as dietary saturated fat, which has been shown to increase intestinal permeability in vivo [22]. In general, more studies on the effect of nutrition on intestinal barrier function and the mechanisms are needed.

## 3. Proteobacteria as a Marker of Dysbiosis and Mediator of Inflammation

In a healthy intestine, Proteobacteria is numerically a minor phyla. Increased abundance of Proteobacteria, particularly the class gamma-Proteobacteria and the family Enterobacteriaceae within it, has been associated with a number of human diseases [23]. As Gram-negative bacteria their outer membrane surface is covered with LPS, which is a complex glycolipid and a major bacterial virulence factor [24,25]. The LPS molecule is composed of three domains; lipid A, core, and O-specific polysaccharides [26]. The lipid A structure, which determines the immunogenicity of LPS, differs among different bacterial species. Most of the Gram-negative bacteria express the hexaacylated LPS, which is one of the most potent agonists of the human innate immune system. LPS triggers a strong proinflammatory reaction and secretion of proinflammatory cytokines from the host cells [27]. Proinflammatory cytokines include interferon-γ (IFN-γ), tumor necrosis factor-α (TNF-α), and interleukin-1β (IL-1β), whereas anti-inflammatory cytokines IL-10 and IL-17 decrease and restore the intestinal permeability [20]. In addition of hexaacylation, tetraacylated and pentaacylated LPS also exist among Gram-negative bacteria. The number of lipid A acyl chains usually correlates well with its ability to induce proinflammatory cytokine production and the hexaacylated forms are the most proinflammatory ones [28]. Consequently, proinflammatory cytokines involved in the intestinal inflammation modify the tight junctions leading to disruption of the intestinal barrier, which plays a role in the pathogenesis of many intestinal diseases, such as inflammatory bowel disease (IBD). 

Due to the highly proinflammatory cell wall structure, Proteobacteria can induce and maintain unspecific inflammation in the intestine without an actual infection if their abundance in the gut microbiota has been increased. The Proteobacteria phylum also includes several notable human pathogens. The high abundance of Proteobacteria has been associated with many disease conditions where intestinal or systemic inflammation or both of these are involved as a key pathoetiological factors. For example, the connection between LPS sustained low-grade inflammation and the development of metabolic disorders has been well established [29]. It has been shown that members of the Enterobacteriaceae family are more abundant in obese people when compared to healthy controls with normal weight. After weight loss, the Enterobacteriaceae population was the most affected family with the relative abundance decreasing from 13% to less than 1% [30]. Furthermore, it was shown that gamma-Proteobacteria were increased in the intestines of patients suffering from nonalcoholic fatty liver disease and nonalcoholic steatohepatitis (NASH) [31]. When comparing the microbiota composition of healthy and obese children, as well as children suffering from NASH, a gradual increase in Proteobacteria has been observed, respectively [32]. Proteobacteria have also been found to be increased in the microbiotas of IBD patients [33,34,35].

Despite the proinflammatory nature of Proteobacteria, it has been well established that there are strain-dependent variations in the immunomodulatory properties. A striking example of this is the Proteobacteria *Escherichia coli* Nissle 1917 (EcN), which is widely used as a probiotic strain [36]. EcN has a specific LPS form, due to a point mutation, which gives it an ability to withstand antibacterial defense mechanisms of blood serum [37]. EcN has been shown to have efficacy in reducing the severity of rotaviral diarrhea and modulating viral immunity in several randomized clinical studies and experimental studies in an animal models [38,39,40,41,42,43]. In addition, it has been shown to very efficiently maintain remission in ulcerative colitis (UC) [44,45,46]. Interestingly, the probiotic effect of EcN is, in part, due to its antimicrobial effect via the production of bacteriocins and inhibition of other *E. coli* strains [47]. Furthermore, EcN interacts with the gut epithelium by inducing the defensin production, strengthening the epithelial layer, decreasing the production of proinflammatory cytokines, and increasing the production of anti-inflammatory cytokines [47]. Thus, EcN seems to counteract the proinflammatory effects commonly induced by Proteobacteria.

## 4. Health-Associated Commensal Bacteria

**Figure 2:**
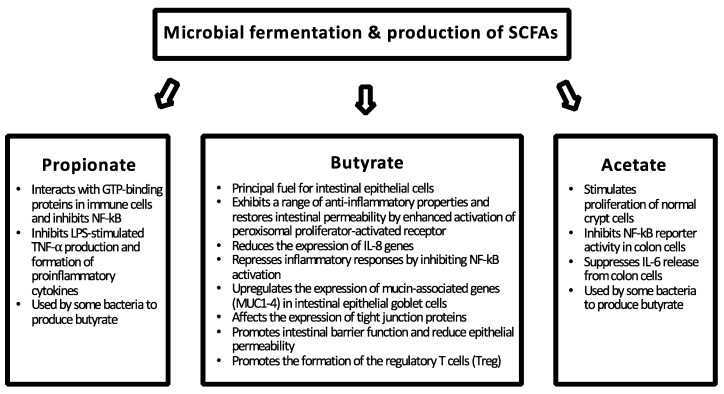
The major short-chain fatty acids (SCFAs) produced by gut microbiota and their health-promoting effects on the gut epithelium [54,55].

A dysbiotic microbiota can be ameliorated with the health-promoting action of next-generation probiotics or boosting the growth and metabolism of beneficial commensals in the colon with fiber-rich food components, especially targeting the butyrate production. The protective effect of commensal bacteria on barrier function is achieved by preventing the colonization of pathogens, interacting directly with the host enterocytes and metabolizing undigested carbohydrates to short-chain fatty acids (Figure 1). Commensal bacteria metabolize dietary fibers and resistant starch to SFCAs, such as butyrate, acetate, and propionate, which serve as a major energy source for the host enterocytes (Figure 2). Dietary fibers and SFCAs have been shown in vivo to suppress intestinal inflammation and colon cancer [48]. Microbial-derived butyrate increases mitochondrial-dependent oxygen consumption in enterocytes, stabilizes the hypoxia-inducible factor (HIF) involved in barrier protection, and induces expression of HIF-target genes that augment barrier function [49]. On the other hand, intestinal homeostasis is also maintained via direct interaction of the microbiota and the host epithelium and immune system, where the recognition of bacterial molecules, such as LPS, by host PRRs plays a major role (Figure 1) [50].

In the following paragraphs, we summarize the recent research results from the promising, cultivable commensal bacteria with health-promoting and anti-inflammatory capacity in the gut. Probiotic characteristics are generally considered strain-dependent and, therefore, cannot be generalized to cover other than the studied strain of the species unless the mechanism of action is known and the strains in question are known to harbor or produce the effector molecules [51,52]. There is a strong evidence of strain-specificity, as well as disease-specificity of probiotic action, which makes it very challenging to authenticate the efficacy of probiotics in clinical trials [52]. Furthermore, it can be difficult to show the beneficial action of probiotics in healthy individuals by recording specific biomarkers rather than symptoms and if the study cohort is not a high-risk population with elevated levels of biomarkers [53]. Thus, all strains of a specific species cannot be expected to exert all described traits described below, but the purpose is rather to give an overview of the beneficial traits found within a species or genus.

### 4.1. Bifidobacterium *spp.*

The genus *Bifidobacterium*, belonging to the phylum Actinobacteria, contains 51 recognized species and 10 subspecies [56], the majority of which originate from human or animal (mammals and bees) digestive tracts, and also few species that have been originally isolated from other sources, such as milk or sewage [57]. For 100 years these bacteria have been considered health-promoting and bifidobacterial strains have been widely used as probiotics along with lactobacilli. The level of knowledge concerning the health-promoting mechanisms of action, as well as the efficacy of bacteria marketed as probiotics varies substantially between strains, which can complicate the comparison and selection of certain strains to specific indications [58]. However, some strains of *Lactobacillus* and *Bifidobacterium* spp. are extensively characterized, also in comparative genomics studies, which makes them good examples and forerunners in the search of next-generation probiotics targeted for specific therapeutic purposes [57,59].

In humans, bifidobacteria are among the founder-species of the infant gut and frequently one of the major taxa or even the most abundant genus of infant gut microbiota [60]. In vaginally born infants, bifidobacteria are found within three days after birth, and after one week they may compose as high as 96.4% of the overall microbiota in breast-fed infants [61,62]. Bifidobacteria seem to be transferred vertically from mother to infant as evidenced by both metagenomic studies and whole genome sequencing of isolated strains from mother-infant pairs [61,63,64]. Duranti et al. [61] showed that strains of *B. longum*, *B. bifidum*, *B. breve*, *B. adolescentis*, *B. dentium*, *B. pseudocatenulatum*, and *B. catenulatum*, i.e., representatives of all major human bifidobacterial species, were shared between the mothers and their infants. The relative proportion of bifidobacteria in children declines after weaning, but stays higher than in adults over the childhood and pre-adolescentis years [65,66]. The abundance of bifidobacteria in adults is estimated to be 1–2% in many countries around the world, and approximately 7% in the Japanese population [66]. The age-related decline in the proportion of bifidobacteria seem to continue until senescence.

Human milk oligosaccharides (HMOs) stimulate the growth of bifidobacteria, but different species and strains have their specific HMO preferences [67]. For example a positive correlation was found between sialylated HMOs and *B. breve* and non-fucosylated/non-sialylated HMOs and the *B. longum* group [67]. Likewise, bifidobacterial species and subspecies have different abilities to degrade glycans of the intestinal mucus layer and plant-derived polysaccharides, which makes some strains better adapted to infant gut, while some are better adapted to adults [68,69,70]. On the other hand, *B. breve* has capabilities to utilize both plant- and host-derived carbohydrates and could, therefore, be called a generalist [71]. Altogether, bifidobacterial genomes are better equipped with genes related to carbohydrate metabolism than average bacterial genomes, and by resource sharing and cross-feeding, bifidobacteria may have large effects on overall gut physiology [71]. In vitro co-culture studies have shown that bifidobacteria have the ability to degrade oligo-fructose and produce acetate, thereby promoting butyrate production of *Faecalibacterium prausnitzii*, *Anaerostipes caccae*, and *Roseburia intestinalis*, by means of cross-feeding [72,73,74]. Detailed knowledge on the metabolic capabilities and cross-feeding chains could open a possibility to modulate the composition and action of gut microbiota in a desired direction by premeditated dietary fiber consumption.

Epithelial adhesion of bifidobacteria is mediated by extracellular structures, e.g., pili or fimbriae and the extracellular polysaccharide (EPS) layer or capsule (Table 1) [57]. Most strains of bifidobacteria have pilus-encoding loci in their genomes. The role of pili in mediating bifidobacterial adhesion to enterocytes, as well as in autoaggregation, was first demonstrated for *B. bifidum* [75]. In a murine model, *B. bifidum* pili evoked TNF-α expression and a significantly lower IL-10 response in cecal mucosa as compared to nonpiliated cells [75]. Later, another type of pili, tight adherence (Tad) pili were discovered in *B. breve* [76]. Interestingly, the genes of *B. breve* Tad-pilus were found to be heavily up-regulated in vivo in a mouse model as compared to in vitro cultivation [76]. Experiments with pilusless mutant strains confirmed the importance of this structure in the colonization and persistence of *B. breve* in the gut. In addition to pili, other surface structures of bifidobacteria such as EPS, also have interesting functional properties. EPS provides acid and bile resistance to bacteria and can act as an immunological silencer by modulating B-cell trafficking and IL-12 production in the epithelium [77]. Furthermore, EPS may play a role in reducing initial colonization and burden of pathogens as evidenced by *Citrobacter rodentium* infection mouse model [77]. 

In addition to extracellular structures, also secreted substances of bifidobacteria can mediate probiotic effects. Acetate, an end product of bifidobacterial fermentation, can give protection against *E. coli* O157 by affecting the regulation of certain genes involved in anti-inflammatory responses and preventing or alleviating damage to intestinal epithelium due to *E. coli* O157-induced cell death and reducing the translocation of toxins from gut to systemic circulation [78]. The resistance to infection was mediated through a higher amount of mouse fecal acetate produced by certain bifidobacterial strains, while the growth rate or the expression of virulence genes of *E. coli* O157 were not affected. The role of acetate was apparent also in the ability of a *B. bifidum* strain to strengthen tight junctions and prevent the disruption of epithelial barrier induced by TNF-α in vitro [79]. Antagonism of cytotoxic effects of *Clostridium difficile* toxins by certain strains of bifidobacteria has been demonstrated with HT29 intestinal epithelial monolayer, where effective strains were able to remove toxins from the medium and preserve the F-actin microstructure and tight junctions between epithelium [80].

Recently, the immunomodulatory ability of various bifidobacterial species was studied with naïve mouse spleen and dendritic cells and immunostimulatory patterns were found to be strain-specific (Table 1) [81]. Interestingly, a strain with a low stimulatory potential in vitro was able to prevent clinical symptoms in a mouse model of dextran sulfate sodium (DSS)-induced colitis, preserve the tight junction protein expression, and protect the epithelial barrier function, while another strain of the same species and with a higher in vitro immunomodulation capacity was not. These examples of bifidobacterial ability to protect the epithelial barrier and interact with the host immunology tells about strain-specific properties and highlights the need of a detailed characterization of selected bacteria and their effects on the molecular level in the search for specific therapeutic properties of the bacteria.

Due to the high proportion of bifidobacteria in the healthy infant gut and the significant reduction of it in the elderly, benefits of bifidobacterial probiotic strains, especially in those two age groups, has raised interest. The effects of probiotic bifidobacteria on the health of pre-term infants is widely studied and several systematic reviews and meta-analyses have found them effective in reducing the risk of necrotizing enterocolitis and sepsis, which are major causes of death among pre-term infants, and in reducing mortality and hospital stays [90,91,92]. In the elderly, bifidobacterial probiotics can improve constipation and enhance cellular immune activity [93,94].

### 4.2. Akkermansia muciniphila

*Akkermansia muciniphila* is a Gram-negative, anaerobic, mucin-degrading bacterium which belongs to the Planctomycetes-Verrucomicrobia-Chlamydia superphylum [95,96]. The type strain *A. muciniphila* MucT was originally isolated from a healthy adult by using gastric mucin as a sole carbon and nitrogen source [95,97]. Later, it was also found to tolerate low levels of oxygen [95,97]. By degrading the complex mucin glycoproteins and due to its ability to produce acetate, propionate and 1,2-propandiol, *A. muciniphila* contributes to the bacterial community cross-feeding [95,98]. Co-culturing of butyrate-producing bacteria with *A. muciniphila* revealed two types of trophic chains, metabolic and cofactor syntrophic interactions. [98]. First, *A. muciniphila* can metabolically support the growth of butyrate-producers by degrading mucin and providing acetate. Second, *A. muciniphila* can use pseudovitamin B_12_ produced by other bacteria for its propionate production. Additionally, *A. muciniphila* produces sulfatases and it may be able to use hydrogen sulfide for cysteine production and, as a result, limit the possible toxicity of sulfate-reducing bacteria [83,96]. 

The abundance of *A. muciniphila* has been found to be reduced in many disease states [99,100,101,102,103]. Studies on metabolic disorders, such as obesity and diabetes, have given the most promising evidence on the beneficial effect of *A. muciniphila* on health [96]. Everard et al. [104] noticed that a daily treatment with live *A. muciniphila* restored the mucus layer thickness in mice on a high-fat diet (HF diet) and reversed the HF diet-induced metabolic disorders. The bacterium also contributed to the production of antimicrobial peptides in the colon as it was found to affect RegIIIγ production [104]. Furthermore, administration of *A. muciniphila* increased the number of mucin-producing goblet cells and anti-inflammatory regulatory T cells (Treg) in mice fed with HF-diet [105]. More recently, the bacterium was shown to protect against atherosclerosis in an Apoe−/− mouse model by attenuating metabolic endotoxemia-induced inflammation through restoration of the gut barrier [106]. In vitro results showing that *A. muciniphila* adheres to intestinal epithelial cell lines and strengthens the cell monolayer support the idea that the bacterium has a special barrier fortifying function in the epithelium [107]. *A. muciniphila* also induces only a low-level proinflammatory stimulation (IL-8) in enterocytes as compared to *E. coli*, which may be beneficial to the gut health as it keeps the immune system alerted [107]. Extracellular vesicles of *A. muciniphila* were also found to have a positive effect on intestinal integrity, and due to their small size they may also reach gut epithelium in places where the mucus layer is firm and impermeable to bacteria [85,108].

Interactions of *A. muciniphila* outer membrane proteins with cells of the human immune system have been studied in vitro [83,109]. These outer membrane proteins are involved in the health-promoting effects of *A. muciniphila* towards the intestinal homeostasis and barrier function (Table 1). The 32 kDa pili-like protein (Amuc_1100), was found to induce IL-1β, IL-6, IL-8, IL-10, and TNF-α production in human-derived peripheral blood mononuclear cells (PBMCs) [83]. Toll-like receptor (TLR) 2 was induced by the purified recombinant Amuc_1100 protein. Furthermore, both *A. muciniphila* and purified Amuc_1100 enhanced trans-epithelial resistance. Interestingly, treatment with Amuc_1100 protein (or pasteurized *A. muciniphila)* lowered body weight and fat mass gain in HF-diet fed mice, corrected HF-diet-induced higher adipocyte diameter and hypercholesterolemia and improved glucose tolerance [84]. Furthermore, Amuc_1100 maintained its activity in stimulating TLR2 after pasteurization, as its melting temperature is 70°C. Thus, Amuc_1100 seems to be responsible for mediating at least part of the beneficial effects of *A. muciniphila*. The first clinical trial suggests that live or pasteurized *A. muciniphila* is safe for use for obese humans and it is a promising candidate for next-generation probiotics [84]. 

### 4.3. Faecalibacterium prausnitzii

*F. prausnitzii* is one of the most abundant bacterial species inhabiting the human intestine, representing 2–15% of the total bacterial load [110,111]. Taxonomically, *F. prausnitzii* belongs to the phylum Firmicutes, class Clostridia, and family Ruminococcaceae with the species currently being the only characterized representative within the genus [112]. The species itself comprises at least two distinct phylogroups based on 16S rRNA sequencing [113]. Metabolically, *F. prausnitzii* is an extremely oxygen-sensitive anaerobe [112]. The bacterium can use various simple sugars as its energy source, whereas the utilization of more complex carbohydrates is strain-specific [114]. The nutrients may be either host- or diet-derived, or they may result from cross-feeding by other gut microbes [114]. The major fermentation products from glucose and acetate by *F. prausnitzii* are formate, d-lactate, and butyrate [112].

*F. prausnitzii* promotes gut health in various ways [114,115]. For instance, it is involved in the feeding of colonocytes, the maintenance of immune homeostasis, and the strengthening of barrier function. It has also been noted that the abundance of *F. prausnitzii* is significantly decreased in several intestinal disorders, such as Crohn’s disease (CD), UC, and colorectal cancer [116,117,118], making it a potential biomarker in the diagnostics of various intestinal disorders. This depletion may result from changes in the environmental parameters, as the bacterium has been shown to be sensitive to environmental changes that accompany intestinal disorders [114]. Replenishment of the diseased gut with *F. prausnitzii* could improve intestinal health making this bacterium a vital part of commensal microbiota [114,115]. In healthy human subjects the consumption of prebiotics, such as inulin (10 g/day) or other dietary fiber (21 g/day), significantly increased the abundance of *F. prausnitzii* [119,120]. Thus, the health-promoting action of prebiotics and fiber could be, in part, mediated by the increase of *F. prausnitzii* and other beneficial commensals.

In respect to health, the substantial production of butyrate by *F. prausnitzii* is of special interest. Butyrate is a major energy source for colonocytes [55] and it also takes part in immune modulation by inhibiting nuclear factor kappa B (NF-κB) transcription factor activation, upregulating peroxisome proliferator-activated receptor gamma (PPARγ), and inhibiting IFN-γ, consequently reducing inflammation (Figure 2) [114]. These anti-inflammatory properties may protect the colon against inflammation and colorectal cancer. As a member of the intestinal ecosystem, *F. prausnitzii* is in constant association with its environment and, therefore, relies on other microbes in order to exhibit its health-promoting effects. It has been proposed that metabolically complementary *F. prausnitzii* and *Bacteroides thetaiotaomicron* modulate the intestinal mucus barrier by affecting the development of goblet cells and the production of mucus glycans [121].

It has been shown that *F. prausnitzii* interacts with the host epithelial cells and attenuates the inflammatory response. According to Sokol et al., *F. prausnitzii* affects the release of cytokines in vitro from PBMCs in a manner that favors the anti-inflammatory cytokine profile (high IL-10/IL-12 ratio) [117]. In the same study, the cell-free culture supernatant of *F. prausnitzii* was shown to reduce the secretion of IL-8 by CaCo-2 cells and to abolish NF-κB activation. Interestingly, the latter was not achieved with butyrate. The experiments on a murine model with acute chemical-induced inflammation showed that both *F. prausnitzii* cells and supernatant reduced the severity of acute colitis and induced increased secretion of anti-inflammatory IL-10 and decreased secretion of proinflammatory cytokines [117]. Furthermore, *F. prausnitzii* cells and supernatant were shown to decrease intestinal permeability by affecting apical junction proteins [122] and enhance the barrier function in murine model with chemical-induced colitis [123]. In addition to butyrate, *F. prausnitzii* produces salicylic acid, which blocks the activation of NF-κB, and, consequently, the production of IL-8 [124]. Furthermore, *F. prausnitzii* produces an anti-inflammatory protein called Microbial Anti-inflammatory Molecule (MAM), whose peptides inhibit the activation of NF-κB in vitro and in vivo (Table 1) [86,125]. The anti-inflammatory properties of MAM produced by *F. prausnitzii* were also shown to inhibit T helper 1 cells (Th1) and Th17 immune responses in chemically-induced colitis models in mice [86]. Although it is evident that *F. prausnitzii* or its components are able to modulate immune responses, many of the effector molecules still remain uncharacterized, emphasizing the need for further studies. Since all the *F. prausnitzii* strains isolated from healthy volunteers showed promising anti-inflammatory properties in vitro [126], the species is a promising target for therapeutic purposes. However, the development of therapeutic supplements utilizing strictly anaerobic bacteria, such as *F. prausnitzii*, is undoubtedly challenging due to the demand of anaerobic conditions and large-scale production. Possible solutions would be freeze-drying the microbial cell biomass, followed by encapsulation or isolating the commensal-derived effector molecules harboring the beneficial effect [127,128].

### 4.4. Roseburia intestinalis

*R. intestinalis* is a Gram-positive, anaerobic, butyrate-producing bacteria originally isolated from the human feces [129]. It belongs to the phylum Firmicutes and family Lachnospiraceae. The genus *Roseburia* includes five characterized species, *R. intestinalis*, *R. hominis*, *R. inulinivorans*, *R. faecis*, and *R. cecicola* [130,131]. The species of genus *Roseburia* are among the most abundant butyrate-producing bacteria accounting for 0.9 to 5.0% of the total microbial load [132]. The abundance of *Roseburia* has been found to be decreased in many intestinal disorders suggesting the bacterium has an important role in maintaining the gut homeostasis, e.g., by producing SFCAs [133]. Significant reduction in the abundance of *Roseburia* spp. and other butyrate and propionate-producing bacteria has been observed in patients with UC [101,134]. Similarly, the abundance of *R. inulivorans* and other butyrate producers has been shown to be decreased in patients with CD compared to healthy controls [135]. Geirnaert et al. studied the microbiota profile of CD patients after supplementation of butyrate-producing bacteria, including *R. hominis* and *R. inulivorans*, using an in vitro system [136]. The mix of six butyrate producers increased the level of butyrate in the dysbiosed bacterial community and improved the epithelial barrier function in an in vitro model, which suggests that butyrate-producing bacteria may have a therapeutic potential as probiotics.

*R. intestinalis* has also been shown to exert anti-inflammatory actions in in vivo and in vitro models. *R. intestinalis* exhibited an anti-inflammatory pattern regarding cytokines in mono-associated mice by inducing increased, anti-inflammatory cytokine IL-22 production and decreased the production of proinflammatory cytokines IFNγ and IL-17 [137]. Similar results were obtained in another study, which demonstrated the inhibited secretion of IL-17 in vivo and in vitro by *R. intestinalis* [138]. In the same study, the bacterium also promoted the differentiation of Treg cells in a colitis mouse model. Furthermore, *R. intestinalis* exerted anti-inflammatory action against LPS-triggered inflammation in enterocytes by promoting the secretion of thymic stromal lymphopoietin (TSLP) and transforming growth factor beta (TGF-β) [139]. *R. intestinalis* colonizes the gut epithelium possibly by using its flagella to penetrate the mucus layer [140]. The flagellin may act as an effector molecule as it has been shown to induce anti-inflammatory effects by upregulating certain IncRNA (Table 1) [87].

### 4.5. Eubacterium hallii

*Eubacterium hallii* is one the major butyrate producers found in the human gut [141]. Quantitatively, the species represents up to 3% of the total bacteria present in the feces of healthy individuals [110]. As for the taxonomy, this obligate anaerobe belongs to the phylum Firmicutes, class Clostridia, and family Eubacteriaceae [142]. The amount of *Eubacterium* spp. have been reported to decrease in patients with UC and CD as compared to healthy individuals, indicating its value in a healthy, balanced microbial community [135,143]. With respect to intestinal health, *E. hallii* could also be involved in detoxification of the most abundant dietary carcinogen 2-Amino-1-methyl-6-phenylimidazo(4,5-b)pyridine by transforming the compound into glycerol-derived conjugate in the colon [144].

As *E. hallii* cannot use complex carbohydrates, it relies on simple sugars or metabolic intermediates as energy sources [145]. The production of butyrate by *E. hallii* has been shown to result from either utilization of glucose, or acetate and lactate [146]. In the human gut, where *E. hallii* is part of complex nutritional webs, this bacterium is presumably cross-fed by other microbes. Co-culture studies have revealed cross-feeding interactions between lactobacilli, bifidobacteria, and butyrate-producing colon bacteria [147,148]. As inulin-type fructans are metabolized into acetate and lactate by lactobacilli and bifidobacteria, *E. hallii* and other butyrate-producing bacteria can use the metabolites in the production of butyrate. The relationship between *E. hallii* and *A. muciniphila* is another example of cross-feeding as these two bacteria exhibit bidirectional syntropy. Belzer et al. [98] has showed that as *A. muciniphila* degrades mucin, it releases oligosaccharides and 1,2-propanediol which *E. hallii* can use for growth. In turn, *E. hallii* produces pseudovitamin B_12_, which *A. muciniphila* can then use to produce propionate. Additionally, other mucin-degrading bacteria, such as *B. bifidum* can provide mucin-derived monosaccharides to *E. hallii*, which is then able to utilize lactose, galactose, and N-acetylglucosamine (GlcNac). The co-culture of these two bacteria has been shown to result in the production of acetate, butyrate, propionate and formate [147,148]. Recently, *E. hallii* was shown to harbor the capability to produce propionate also independently and to stimulate its production in other microbes [149].

### 4.6. Bacteroides *spp.*

*Bacteroides* species belong to the major phylum Bacteroidetes. The genus *Bacteroides* contains the most predominant species of Bacteroidales order in the human intestinal tract [150]. *Bacteroides* species are adapted to colonize the harsh environment of the intestine with different mechanisms, such as oxygen toleration using cytochrome bd oxidase, metabolizing a variety of diet- and host-derived polysaccharides, and extensive expression of cell surface structures [151]. One of the most abundant species, *Bacteroides fragilis*, a Gram-negative obligate anaerobe, exhibits both beneficial and pathogenic actions for the host [152,153]. The health-promoting properties of the species within this genus have been recognized relatively recently with *B. fragilis* being the best studied representative.

*B. fragilis* produces an immunomodulatory molecule called polysaccharide A (PSA), which activates T-cell-dependent immune responses involved in the gut homeostasis (Table 1) [154]. The bacterium is capable of niche-specific mucosal colonization in the gut, which is PSA-mediated by activating Treg cells through TLR2 signaling to suppress Th17 responses and, thus, inducing mucosal tolerance [155]. Specifically, PSA has been shown to protect from *Helicobacter hepaticus* induced colitis in mice suggesting it has immunomodulatory capacity and is a potential therapeutic mechanism to the host [88]. Moreover, this symbiotic surface factor has been shown to decrease IL-1β-induced IL-8 cytokine levels in human fetal enterocytes, necrotizing enterocolitis enterocytes, and in fetal mouse small intestine through the stimulation of TLR2 and TLR4 receptors [156]. In the DSS-induced colitis mouse model, *B. fragilis* inhibits the expression of proinflammatory cytokines IL-6 and TNF-α and enhances the anti-inflammatory IL-10 expression, and thereby ameliorates colitis symptoms [88,157]. In another mouse model, *B. fragilis* enforces epithelial barrier by correcting intestinal permeability, tight-junction alterations and cytokine expression [158]. In addition, *B. fragilis* affects the host immune system by enhancing the phagocytic function of macrophages and polarizing them into the M1 phenotype, which promotes Th1 response [159].

*B. fragilis* communicates with the host immune system, e.g., via its PSA, which is delivered to dendritic cells packed in outer membrane vesicles (OMVs) released from the bacterial surface (Table 1) [89]. Secretion of these signaling molecules enables *Bacteroides* spp. to interact with the intestinal epithelium, which would be otherwise inaccessible due to the thick mucus layer [160]. *Bacteroides vulgatus*, which has been shown to protect against *E. coli* -induced colitis in monocolonized mice, and also produces OMVs and regulates immune responses in the host [160,161]. In addition to *B. fragilis*, other less studied *Bacteroides* spp. are also capable of producing multiple capsular polysaccharides making their surface structures more diverse [162]. Different *Bacteroides* spp. isolated from fecal microbiota transplantation (FMT)-donor were capable of attenuating LPS-induced inflammation in vitro, which indicates that they may exert anti-inflammatory action in the intestine [163]. However, the attenuation capacity is not adhesion-dependent suggesting the bacteria may secrete effector molecules responsible for the interaction with the enterocytes. The currently used probiotics on the market may not colonize the intestinal tract in the long-term. Many of the probiotic strains have been isolated originally from other niches than the human gut, which could explain their poor colonization capacity. For this reason, these next-generation probiotics are of interest as they could possibly colonize the gut more efficiently, being adapted to the intestinal conditions. In fact, results from FMT-studies suggest long-term colonization of commensal donor species, including *Bacteroides* spp., in the recipients [164,165].

## 5. Conclusions

Certain intestinal bacteria, like those discussed above, reinforce the host epithelium and give cross-tolerance to LPS, which counteracts or decreases the toxic effects of LPS in dysbiosis. Furthermore, these commensal gut colonizers produce immunoregulatory effects via secreted metabolites, such as SCFAs, especially butyrate. *Roseburia*, *Faecalibacterium*, and *E. hallii*-related bacteria are the most abundant butyrate-producers in the human gut [132]. Moreover, cross-feeding chains have been established between bifidobacteria, *F. prausnitzii* and *R. intestinalis*, which enhance the butyrate production in the colon. Additionally, *A. muciniphila* takes part in the cross-feeding by degrading complex mucin glycoproteins to oligosaccharides, which *E. hallii* and other butyrate producers, in turn, are able to use for growth and, thus, the production of butyrate. The importance of butyrate producers to gut health is well established, since these bacteria are significantly reduced in many intestinal diseases. *Bacteroides* species, on the other hand, exert different mechanisms to enhance the gut homeostasis, e.g., secreting immunomodulatory effector molecules. The *B. fragilis*-produced PSA molecule is an exciting discovery in this field. The mechanisms of action of the potential next-generation probiotics at the molecular level are still largely unknown, but the knowledge is increasing rapidly.

Dietary changes and the use of antibiotics associated with intestinal diseases also modulate the microbiota composition, which plays a part in the depletion of essential health-promoting commensals in the colon and, thus, contributes to unbalanced microbiota composition. The most drastic example of disturbed microbial ecosystem and function by antibiotics and, on the other hand, its restoration by bacteriotherapeutic supplementation, is recurrent *Clostridium difficile* infection and its treatment with fecal microbiota transplantation [164]. Considering the beneficial effects of the commensal species in fortifying intestinal barrier function and immunoregulation discussed above, it seems justified to consider strategies to promote their presence and activity in the human gut. This could be achieved by *de novo* introduction of the species into the gut in the form of probiotic or bacteriotherapeutic preparations or by stimulating the growth of the species in the gut by dietary means, or the combination of these two [128,166]. As a promising example, the treatment with *A. muciniphila* has been shown to counteract high fat diet -induced complications and gut barrier dysfunction in mice and the bacterium is tested safe for humans [84,104,105]. The dietary approaches include adequate dosages of fiber and prebiotics, whose beneficial effects are transmitted to the host particularly through bacterial fermentation and the health-promoting action of commensals that are stimulated by these compounds [167]. Interestingly, the microbiota composition and presence of certain key commensal species seem to affect the individual’s fiber fermentability capacity, as was discovered in a feeding study where individuals lacking *Ruminococcus bromii* had reduced fermentability (20–30% compared to 100%) of resistant starch [168]. Within prebiotics, inulin-type fructans and oligosaccharides mimicking HMOs have already shown promising efficacy in modulating the microbiota composition and metabolism, in which cross-feeding has a central role at the gut ecosystem level.

In the future, increased knowledge on the cross-feeding chains within microbiota combined with the affordable profiling of individual’s microbiota will open exciting and efficient possibilities in personalized nutrition to modulate the composition and action of gut microbiota in the desired direction.

## Figures and Tables

**Table 1:** Known effector molecules of the selected commensal bacteria and their effect on intestinal health. All in vivo studies were mice studies.

Organism	Effector Molecule	Mediated Effect	Study Type	Reference
*B. breve* UCC2003	IVb tight adherence (Tad) pili	Host colonization and persistence mechanism	in silico, in vivo	[76]
*B. breve* UCC2003	Surface exopolysaccharide (EPS)	Acid and bile tolerance, immunomodulation, protection against pathogen colonization and burden	in silico, in vitro, in vivo	[77]
Several bifidobacterial strains	Sortase-dependent pili	Adhesion to host mucus components	in silico, in vitro, in vivo	[82]
*B. bifidum* PRL2010	Sortase-dependent pili	Autoaggregation, evoked TNF-α expression and a significantly lower IL-10 response	in vitro, in vivo	[75]
*B. longum* ssp. *longum* CCM 7952	Ligands for NOD2	Reduced clinical symptoms in a colitis mouse model, preserved expression of tight junction proteins	in vitro, in vivo	[81]
*A. muciniphila* MucT	Amuc_1100 (OM pili-like protein)	Induction of cytokine production, interactionwith TLR 2, improves gut barrier	in vitro	[83,84]
*A. muciniphila ATCC BA-835*	Extracellular vesicles	Improves intestinal barrier integrity, improved body weight & glucose tolerance	in vitro, in vivo	[85]
*F. prausnitzii A2-165*	Microbial anti-inflammatory molecule (MAM)	Peptides of MAM inhibit the activation of NF-κB, ameliorate colitis symptoms in mice	in vitro, in vivo	[86]
*R. intestinalis DSMZ-14610*	Flagellin	Upregulation of IncRNA, alleviation of colonic inflammation	in vitro, in vivo	[87]
*B. fragilis NCTC 9343*	Polysaccharide A	Immune activation, elicits cytokine production, protects and treats colitis in an animal model	in vitro, in vivo	[88]
*B. fragilis 323-J-86*	Outer-membrane vesicle	Transports PSA molecule to immune cells	in vitro, in vivo	[89]
